# The Left Hand Second to Fourth Digit Ratio (2D:4D) Is Not Related to Any Physical Fitness Component in Adolescent Girls

**DOI:** 10.1371/journal.pone.0059766

**Published:** 2013-04-02

**Authors:** Maarten W. Peeters, Katrijn Van Aken, Albrecht L. Claessens

**Affiliations:** 1 Department of Kinesiology, KU Leuven, Leuven, Belgium; 2 Department of Rehabilitation Sciences, KU Leuven, Leuven, Belgium; University of Turku, Finland

## Abstract

**Introduction:**

The second to fourth-digit-ratio (2D:4D), a putative marker of prenatal androgen action and a sexually dimorphic trait, has been suggested to be related with fitness and sports performance, although results are not univocal. Most studies however focus on a single aspect of physical fitness or one sports discipline.

**Methods:**

In this study the 2D:4D ratio of 178 adolescent girls (age 13,5–18 y) was measured on X-rays of the left hand. The relation between 2D:4D digit ratio and multiple aspects of physical fitness (balance, speed of limb movement, flexibility, explosive strength, static strength, trunk strength, functional strength, running speed/agility, and endurance) was studied by correlation analyses and stepwise multiple regression. For comparison the relation between these physical fitness components and a selected number of objectively measured anthropometric traits (stature, mass, BMI, somatotype components and the Bayer & Bailey androgyny index) are presented alongside the results of 2D:4D digit ratio.

**Results:**

Left hand 2D:4D digit ratio (0.925±0.019) was not significantly correlated with any of the physical fitness components nor any of the anthropometric variables included in the present study. 2D:4D did not enter the multiple stepwise regression for any of the physical fitness components in which other anthropometric traits explained between 9,2% (flexibility) and 33,9% (static strength) of variance.

**Conclusion:**

Unlike other anthropometric traits the 2D:4D digit ratio does not seem to be related to any physical fitness component in adolescent girls and therefore most likely should not be considered in talent detection programs for sporting ability in girls.

## Introduction

The ratio of the length of the second (index) finger to the length of the fourth (ring) finger (2D:4D) ratio represents an individual difference variable putatively related to prenatal gonadal hormonal exposure. A lower 2D:4D is indicative of relatively higher prenatal testosterone than estrogen levels and a number of studies have investigated the associations between 2D:4D and sporting ability or physical fitness [1]–[19]. The hypotheses set up in these studies were that a lower digit ratio is related to better sport abilities or better motor performance, in both males and females. Overall, results of the studies are not consistent and in part difficult to compare because of the use of different procedures for measuring the digit lengths. In some studies where both sexes are included the relation is found in both men and women [4], [6], [12], [16], although in the study of Hönekopp et al. [6] the relation in women was significant only for the left hand 2D:4D and not for the right hand 2D:4D and vice versa in men. Other studies found a significant relation in men but not in women [7], [17] and vice versa [14]. Fink et al. [3] reported a relation between 2D:4D and hand-grip strength in men, but this was not replicated in men [15] nor found in women [13], [15], [17]. A number of studies found the relationship between sporting ability and digit ratio in both right and left 2D:4D [1], [7] in right 2D:4D but not in left 2D:4D [2], [3], [14] or vice versa [11] with the mean of right and left 2D:4D [10] or neither with right nor left 2D:4D [5], [15], or only with the difference between right and left 2D:4D [5]. In a meta-analysis Hönekopp and Schuster [20] reported that for the association between 2D:4D digit ratio and athletic prowess neither hand out-predicted the other. Also, in many studies sporting abilities were not measured objectively, but based on information reported by the participants themselves [10], [12]. In addition, many of the published studies on sporting ability and 2D:4D focused on one aspect of physical fitness, mostly static strength as measured by handgrip strength [3], [13], [15]–[17], or a number of different sports [1], [2], [7], [8], [10], [14], [18], [19] and only a few studies report data for adolescents [6], [9], [18], [19]. Potential anthropometric covariates, if present, are mostly limited to (self reported) stature and mass. Physical fitness however cannot be assessed by a single test and consists of multiple distinct aspects such as balance, flexibility, speed of limb movement, sprinting speed, endurance and static, explosive, functional, and trunk strength as is evidenced by factor analyses e.g. [21]. Physical fitness test-batteries have been developed to cover the range of physical fitness components in field-tests allowing to reliably asses the physical fitness of large numbers of subjects. To our knowledge currently there are no studies reporting the potential relation between the 2D:4D digit ratio and multiple aspects of objectively assessed physical fitness in adolescent girls.

## Materials and Methods

### Samples

Data from the 178 girls used in the present study are part of the Leuven Growth Study of Flemish Girls (LGSFG) [21]. The LGSFG (n = 9414 girls between 6–18 years) was a representative sample of the Flemish population in the academic year of 1979–1980. The sampling was done with the guidance of the Statistical Service of the Belgian Ministry of education using a multistage procedure. In the first stage a proportionate stratified sample with schools as the primary sampling cluster was selected. This sample included 43 primary schools and 45 secondary schools. In the second stage, all students in a single classroom at each grade level were selected within each school [21]. From this study a sample of 178 secondary school girls between 13 and 18 years of age was selected. Only girls with the following conditions were selected: (1) girls for whom an X-ray of the left hand was available; and (2) girls must be sedentary, i.e. not practice any sports apart from the mandatory 2 hours/week of physical education classes in school. This was done to minimize the potentially confounding influence of differences in physical activity on physical fitness which may mask a possible relation between 2D:4D and physical fitness. The mean chronological age of the selected girls was 15.7+1.3 years, varying from 13.2 to 18.4 years. This sample contains all 129 girls that were used to compare the digit ratio of world-class gymnasts to that of sedentary girls in a previous study in which sedentary girls were age-matched to elite gymnasts [18]. The Leuven growth study of Flemish Girls was approved by the Medical Ethics Committee of the Institute of Physical Education of the Catholic University of Leuven. Written informed consent was given by the school authorities at the national and local levels, and by the parents of the children [21]. Anthropometric characteristics of the sample are presented in table 1.

**Table 1:** Descriptive statistics.

	Variable	Mean±SD (range)
	Chronological age (yrs; n = 178)	15.7±1.3 (13.2–18.4)
	Skeletal age (yrs; n = 178)	15.0±1.2 (12.3–16.0)
Anthropometry	2D:4D (n = 178)	0.925±0.019 (0.865–0.969)
	Stature (cm; n = 178)	162.2±6.4 (140.4–185.2)
	Mass (kg; n = 178)	54.1±8.9 (30.3–110)
	BMI (kg/m^2^; n = 178)	20.6±3.0 (13.8–42.9)
	Endomorphy (n = 177)	3.9±1.2 (1.8–8.0)
	Mesomorphy (n = 178)	3.0±1.1 (0.2–10.0)
	Ectomorphy (n = 178)	3.0±1.3 (0.1–7.8)
	Iandr (n = 178)	76.6±4.7 (67.3–103.2)
Physical fitness components		
Balance	Flamingo Balance (n attempts; n = 170)	12.7±6.4 (2–30)
Speed of limb movement	Plate Tapping (n taps in 20 s; n = 176)	79.9±9.0 (54.0–104.0)
Flexibility	Sit and Reach (cm; n = 176)	24.6±7.2 (4.0–44.0)
Explosive strength	Vertical Jump (cm; n = 171)	32.3±6.5 (18.0–70.0)
Static strength	Arm Pull (kg; n = 176)	41.2±9.0 (21.0–62.0)
Trunk Strength	Leg Lifts (n in 20 s; n = 170)	15.1±3.9 (0.0–23.0)
Functional strength	Bent Arm Hang (s; n = 172)	10.4±9.9 (0.0–64.1)
Running speed, agility	50 m Shuttle Run (s; n = 167)	22.0±1.5 (18.4–29.5)
Endurance	480 m Shuttle Run (s; n = 100)	164.5±12.5 (143.3–201.4)

### Physical Fitness Components

The physical fitness tests used in the present study are based on a factor analyses in 402 Flemish girls prior to the start of the LGSFG. Tests were selected for test-retest reliability and having a high loading on a single factor after orthogonal and oblique rotations. A detailed description of the construction of the test battery and selection of the tests is given by Simons et al [21]. Later many of these tests or modifications thereof were included in the Eurofit test battery, which includes the same fitness components [22]. The components and tests used in the present study are shown in table 1. Briefly a high score represents higher fitness for Plate tapping (n taps in 20 seconds), Sit and reach (cm), vertical jump (cm), arm pull (kg), leg lifts (n in 20 seconds), and bent arm hang (seconds), while a lower score represents a better performance for flamingo balance (n attempts to accumulate 1 minute of standing on the 3 cm wide balance beam) 50 m shuttle run, and 480 m shuttle run. The latter shuttle run test is less commonly used but was shown to be a practical and reliable indoor field test of endurance in Flemish boys and girls [23]. This test is similar to the 50 m shuttle run procedure yet for this test the subject has to run 24*20 m vs 10*5 m in the 50 m Shuttle run.

### Anthropometric Dimensions

In order to compare the relative strength of the potential relation of 2D:4D with physical fitness to that of other more common antropometric the following measurements were selected from the database: mass; height; biacromial and bicristal breadths; humerus and femur widths; biceps and calf girths; and triceps, subscapular, supraspinale, and calf skinfolds. All bilateral measurements were taken on the left side of the body. Body mass index (BMI) was calculated as mass (kg)/height (m^2^). All measurements were taken by well-trained observers according to the measuring procedures as described by Claessens et al. [24].

### Body Ratio of Sexual Dimorphism

The *androgyny index* : the Bayer-Bayley ratio (Androgyny Index, IANDR) [25] relates the breadth of the hips (lower trunk, bicristal breadths) to that of the shoulders (upper trunk, biacromial breadth).

IANDR = (bicristal breadth-cm/biacromial breadth-cm) × 100.

On average the ratio is higher in girls than in boys at virtually all ages during childhood and adolescence, and this difference persists into adulthood.

This index is a useful indicator of sex differences in the proportional relationship of the shoulders and hips [25]–[27].

### Somatotype

The three somatotype components endomorphy, mesomorphy and ectomorphy were anthropometrically determined according to the Heath-Carter technique. The Heath-Carter anthropometric somatotype is calculated from 10 dimensions: mass; height; triceps, subscapular, supraspinale, and calf skinfolds; humerus and femur widths; and biceps and calf girths. Endomorphy is derived from the sum of three skinfolds (triceps, subscapular, supraspinale) adjusted for height. This component describes the relative degree of fatness of the body. Mesomorphy is derived from humerus and femur widths, biceps and calf circumferences corrected for the triceps and calf skinfolds respectively, and height. The four limb measurements are adjusted for height. This component expresses the relative degree of muscle, bone and connective tissue. Ectomorphy is based on the somatotype ponderal index: height (cm) divided by mass (kg)^1/3^. This component characterizes the degree of linearity, slenderness, and fragility of body build, with poor muscular development, and a predominance of surface area over body mass. For a detailed description how the three components were calculated reference is given to Claessens et al. [26].

### Skeletal Age

An X-ray of the left hand and wrist of each subject was taken for the assessment of skeletal maturity. An Elinax 60 (62 kV, 15 mA) assembled in a portable apparatus was used. Skeletal age (Skel.age) was estimated using the radius-ulna-short bone protocol in the Tanner-Whitehouse II method. With this method, the maturity status of the radius, ulna, the first, third, and fifth metacarpals, and phalanges of the first, third and fifth fingers are rated, the bone-specific scores are summed into a maturity score, and the maturity score is converted to a skeletal age. For more detailed information reference is given to Claessens et al. [26].

### Measuring 2D and 4D Lengths

#### Measuring procedure

Radiographs from the left hand of all the subjects were available to measure and calculate 2D:4D. Only left hand radiographs were available for the present study because these were taken originally to determine skeletal age, which according to the Tanner-Whitehouse II method is based on left-hand radiographs. The lengths of the second and fourth finger were measured from the proximal end of the proximal phalanx to the distal tip of the distal phalanx using a caliper accurate to 0.1 mm (John Bull British Indicators LtD, England). Digit lengths of each sample were measured by two raters. The mean of the two raters was taken as the final measurement.

#### Reliability study

Before measuring all X-rays, a reliability study was conducted by three different raters. This study consisted of the measurement of thirty left-hand X-rays twice by each rater in a test and re-test manner. Interobserver measurement repeatabilities for 2D:4D ratios were assessed with intraclass correlation coefficients (ICC). The ICC for interrater reliability showed a reliability of 0.98. Anova did not show any significant difference for 2D:4D between the raters. The technical error of measurement for all raters was <0.001 for 2D:4D. It therefore can be concluded that all measurements of 2D:4D were measured reliable.

### Statistical Analyses

Normality of all variables was verified by Kolmogorov Smirnov test. Where necessary log transformations or SQRT-transformation were applied to obtain or approach normal distribution of the variables prior to analysis. Partial Pearson product moment correlations between 2D:4D, anthropometric dimensions and physical fitness components were calculated in order to obtain the correlation without the potentially confounding influence of chronological age or skeletal age on physical fitness components and anthropometric dimensions. Multiple stepwise regression analyses (PROC REG SAS 9.1.3) were performed with physical fitness components as the dependent variables and 2D:4D and the other anthropometric dimension as the independent variables. Tolerance and Variance Inflation were checked to detect further potential issues with multicollinearity. The Statistical Analysis System program 9.1.3 (SAS Institute, Cary, NC, USA) was used to analyze the data.

## Results

Results from the partial correlation analyses are presented in table 2. Correlations between 2D:4D and physical fitness components were not significant ranging between −0.07 (Vertical jump) and 0.13 (Flamingo balance). With the exception of the sit and reach test, all physical fitness components were correlated significantly with two or more of the other 7 anthropometric measurements with correlations being strongest for static strength, functional strength and endurance. Results including skeletal age instead of chronological age in the partial correlations were similar (results not shown). 2D:4D was not significantly correlated with skeletal (r = 0.07) nor chronological age (r = −0.01), which is consistent with the establishment of digit ratio in prenatal development. 2D:4D was not significantly correlated with any of the other anthropometric traits in the study ranging between −0.14 for stature and +0.09 for BMI (results not shown).

**Table 2:** Pearson correlations between anthropometric characteristics and physical fitness components in Flemish adolescent girls.

	FBA	PLT	SAR	VTJ	ARP	LEL	BAH	SHR50	SHR480
2D:4D	0.13	0.06	0.06	−0.07	−0.08	0.03	−0.06	0.05	−0.01
	−0.03;0.27	−0.08;0.22	−0.08;0.21	−0.21;0.09	−0.22;0.07	−0.12;0.18	−0.21;0.09	−0.10;0.20	−0.21;0.18
Stature	−0.12	0.22*	−0.07	0.17*	0.31*	−0.20*	0.01	−0.09	−0.20
	−0.27;0.03	0.07;0.35	−0.21;0.08	0.02;0.31	0.17;0.44	−0.33; −0.05	−0.14;0.16	−0.23;0.06	−0.38;0.00
Mass	0.18*	0.19*	0.05	−0.08	0.49*	−0.12	−0.39	0.14	0.14
	0.03–0.32	0.04;0.33	−0.10;0.20	−0.23;0.07	0.39;0.59	−0.26;0.04	−0.51; −0.25	−0.02;0.28	−0.06;0.33
BMI	0.28*	0.08	0.10	−0.19*	0.37*	−0.02	−0.44	0.21*	0.30*
	0.13;0.41	−0.07;0.23	−0.05;0.24	−0.33; −0.04	0.23;0.49	−0.17;0.13	−0.56; −0.31	0.06;0.35	0.10;0.47
Endo	0.28*	−0.02	−0.05	−0.37*	0.11	−0.17*	−0.51*	0.32*	0.42*
	0.13;0.41	−0.17;0.13	−0.20;0.10	−0.49; −0.23	−0.04;0.25	−0.31; −0.02	−0.61; −0.39	0.17–0.45	0.24;0.59
Meso	0.17*	−0.01	0.13	−0.10	0.25*	0.08	−0.28*	0.14	0.24*
	0.02;0.31	−0.16;0.14	−0.02;0.27	−0.25;0.05	0.11;0.38	−0.07;0.23	−0.41; −0.13	−0.02;0.28	0.04;0.41
Ecto	−0.27*	0.01	−0.13	0.21*	−0.25*	−0.08	0.41*	−0.11	−0.33*
	−0.41; −0.13	−0.13;0.16	−0.28;0.02	0.06;0.35	−0.39; −0.11	−0.23;0.07	0.27;0.53	−0.27;0.03	−0.49; −0.14
Iandr	0.15	−0.02	−0.16*	−0.19*	−0.02	−0.20*	−0.23*	0.32*	0.08
	−0.00;0.29	−0.17;0.13	−0.30; −0.01	−0.33; −0.04	−0.17;0.13	−0.34; −0.05	−0.37; −0.08	0.18–0.46	−0.12;0.28

* = p<0.05; First line = partial correlations with chronological age partialled out; second line: 95%Confidence Intervals on the partial correlations; Endo = endomorphy; Meso = mesomorphy; Ecto = ectomorphy, Iandr = Bayer Bayley ratio; FBA = Flamingo Balance, PLT = Plate tapping; SAR = Sit and reach; VTJ = Vertical Jump, ARP = Arm pull; LEL = Leg lifts BAH = Bent arm hang, SHR50 = 50 m shuttle run; SHR480 = 480 m shuttle run.

The multiple regression analyses are presented in table 3. Anthropometric dimensions explained between 9.2% (flexibility) and 33.9% (static strength) of the variation in physical fitness components, however 2D:4D never entered the regression significantly in any step of the analyses and consequently was never included in the final regression models. Endomorphy (7 times) and chronological age (5 times) most often entered the regression models significantly. The minimal tolerance was 0.34 and the maximal VIF was 2.92 for endomorphy in the multiple regression for SHR50 suggesting there were no multicollinearity problems in the regression analyses.

**Table 3:** Stepwise multiple regression of relation between selected antropometric characteristics and Physcial fitness components in adolescent Flemish girls.

Dependent variable	Intercept	Regression Coefficient	Independent variables	R^2^ *100 (cumulative)
Flamingo balance	7.36	0.22	Endomorphy	6.35
		−0.12	Chronological age	10.15
		−0.02	Stature	11.63
Plate tapping	4.43	0.03	Chronological age	11.58
		0.01	Stature	15.88
Sit and reach	28.25	−1.98	Endomorphy	4.91
		−2.05	Ectomorphy	7.78
		0.65	Chronological age	9.17
Vertical jump	0.54	−0.04	Endomorphy	12.25
		0.02	Chronological age	22.52
		0.50	Mass	29.42
Arm pull	−64.31	105.78	Mass	24.96
		−2.68	Endomorphy	33.03
		−35.37	Iandr	33.89
Leg lifts	20.15	−1.63	Endomorphy	8.36
		−1.32	Ectomorphy	12.75
		0.34	Chronological age	14.05
Bent arm hang	5.50	−0.68	Endomorphy	26.20
50 m shuttle run	15.59	−7.71	Mass	10.20
		0.75	Endomorphy	19.50
		8.48	Iandr	22.48
		0.23	Ectomorphy	24.49
480 m shuttle run	261.92	−73.56	Mass	15.75
		7.50	Endomorphy	22.70

Iandr = Bayer-Bayley ratio. Variables were transformed prior to analyses to obtain or approach normal distribution when necessary. Vertical jump, mass, and iandr were Log transformed, flamingo balance, plate tapping, bent arm hang and mesomorphy were square –root transformed.

## Discussion

The results of the present study suggest that in adolescent girls 2D:4D is not related to any of the physical fitness components which are usually included in large scale (growth) studies of physical fitness, physical activity and health and their determinants. It is difficult to compare the present results to previous studies because previous studies have mostly focused on single components of physical fitness and in general do not include adolescent girls. Also most studies do not use radiographs to measure the digit lengths, such that the soft tissue in theory could also partly confound the comparison with the present results. It has been shown however that digit ratio’s based on radiographs, while producing lower mean values, are correlated with direct hand measurements and photocopies [28] and e.g. Paul et al. [10] reported a significant association between radiograph-based 2D:4D digit ratio and running level in adult women.

To our knowledge only a study by Honekopp et al [6] included late adolescent girls (16.9±0.6 yrs) and found a significant correlation between right (r = −0.17) and left (−0.16) hand digit ratios and physical education grades. In a stepwise regression only right hand 2D:4D remained in the solution in their study. In a second part of their study they studied the association between a compound fitness score and 2D:4D, controlling for self –reported BMI and Physical Activity in female university students (20.1±1.9 years). A significant correlation between PF and left 2D:4D (r = −0.32) but not with right hand 2D:4D (r = −0.16) was reported and this association was also confirmed in a multiple regression analysis in which left hand 2D:4D and hours of exercise together explained 18% of variation in the PF-score [6]. These latter results are not supported by the present study. This may be related to the age difference between both studies and the use of a different test to assess PF. In the study by Hönekopp et al. [6] principal component analyses yielded a single factor solution suggesting that their six tests underlie a single construct i.e. ‘physical fitness’, whereas the tests selected for the present study were selected based on a factor analysis with orthogonal and oblique rotations of 24 fitness tests to obtain independent factors and select tests that show the lowest intercorrelations and hence evaluate distinct components of physical fitness [21] in order to confirm that ‘physical fitness’ consists of multiple components.

When looking at the separate components, to our knowledge only static strength, running speed and endurance have been studied in their relationship with 2D:4D, and none of these studies include adolescent girls [3], [5], [8], [9], [13], [15]–[17]. The other 6 components of the present study have not been evaluated previously for their potential association with 2D:4D digit ratio, and hence this study is the first to report that these association do not appear to exist for balance, speed of limb movement, flexibility, explosive strength, trunk strength, and functional strength, although further studies may be needed to confirm the present findings in other groups. The component studied most frequently is static strength which is evaluated by the handgrip test [3], [13], [15]–[17]. A different test for static strength (Arm Pull) was used in the present study however in Flemish girls this factor loaded highly on the same factor as the handgrip test and therefore evaluates the same underlying physical fitness component [21]. In men significant relations between handgrip strength and 2D:4D were reported [3], [16], [17] although not consistently [15]. In women no association was found between static strength and 2D:4D [13], [15], [17] except in elderly (73.72±6.23 y) women [16]. Although our results for static strength are mainly consistent with previous studies in women we feel some caution may be warranted on the appropriateness of the hand grip-test in evaluating a potential relationship between 2D:4D digit ratios and static strength: for this test the dynamometer is adjusted for the length of the first phalange of the middle finger. A certain ratio of the second and fourth digits may either be beneficial or detrimental to performance in this test from a biomechanical point of view such that a potential relation between the static strength component and digit –ratios is either spuriously inflated or conversely masked by the use of hand-grip tests to evaluate static strength. Unfortunately the present study does not include hand-grip data to allow an objective evaluation in this matter.

The second fitness component that has been previously evaluated is endurance [5], [7], [8]. The strongest associations between physical fitness and digit ratio has been reported for endurance running in both adult men and women, explaining about 25% of the variance in endurance running [8]. However in ergometer rowing performance, which requires the need for high power output in addition to a well developed cardiovascular system, the relation was lower, explaining 13,3% and 6.4% of performance for right and left 2D:4D respectively in men, while it was not significant in women [7]. Finally a recent study in 41 adolescent boys (13.9±1.3 years) showed no significant relation between maximal oxygen uptake and right nor left 2D:4D [5], which may be considered to be consistent with the present findings in adolescent girls. However in their study Hill et al [5] even found a positive trend (p = 0.15) for left hand 2D:4D suggesting a high digit ratio to predict high VO2max. They also reported opposite non-significant correlations between left and right hand 2D:4D for velocity on the treadmill at VO2max and peak lactate, which resulted in a significant association between right minus left 2D:4D and these endurance performance variables, which was also confirmed in a multiple regression analysis [5]. Our data however does not contain right hand digit-ratios to explore the existence similar association in girls.

A final component that is reported in the literature relating digit ratio to performance is sprinting speed. In a study by Manning et al [9] showed that in 10–17 year old boys right (r = −0.14) and left (r = −0.15) 2D:4D digit ratio was significantly yet weakly associated with 50 m sprint performance after controlling for age, BMI and a maturation index. These findings were not replicated in the present study in girls. Apart from the sex difference between these studies the slightly different test (50 m shuttle run) may contribute to not finding similar results for running speed: in the 50 m shuttle run subjects have to stop and turn every 5 m (times 10) and in the study by Manning the subjects were allowed to run 50 m in a straight line. Split times were all related to digit ratio except for the first (at 10 meters) [9]. This however may also be related to reaction times, which are not an issue after every ‘stop and turn’ of the 50 m shuttle run used in the present study.

In the present study we also correlated several anthropometric dimensions other than the 2D:4D digit ratio to physical fitness components. It is beyond the scope of this paper to provide an in-depth discussion of these associations. They were merely presented to offer the reader a sense of the magnitude of correlations and associations of other anthropometric characteristics with physical fitness. The explained variance by this limited number of anthropometric characteristics ranged between 9.2% for flexibility and 33.4% for static strength. Endomorphy, which can be considered a proxy for %fat, was the most frequent ‘predictor’ of performance (table 3). For comparison in a homogeneous sample of world-class gymnasts up to 45% of the scores at the 1987 World Championships could be explained by an extensive battery of anthropometric measurements. Endomorphy by itself explained 35,7% of variation in the final score at the championships [29] while digit ratio did not enter the multiple regression significantly [19]. Although these were substantial percentages, the relation with anthropometry by itself was not deemed sufficient to allow prediction of performance scores at an individual level. The weak yet at times significant associations between 2D:4D and fitness and performance reported in the literature (mostly in adults) and which could not be confirmed in the present study in adolescent girls, do not support the use of 2D:4D in a talent detection framework. This is especially true since talent detection in sports is an issue in childhood and adolescence and not in adults.

For the present study it was decided to include only sedentary girls to avoid the potentially confounding effect of variation in physical activity and hours of exercise on the fitness components, which may mask the relation between 2D:4D and performance. While we consider this a strength of our study it might also be perceived as a limitation: it has been shown that 2D:4D is also related to training frequency in endurance athletes [8]. However both training frequency and 2D:4D entered the regression suggesting their effect on endurance performance was at least in part independent. Hönekopp et al [6] found similar results in women for their PF compound score yet suggested that in males the association between 2D:4D may at least in part be mediated by exercising habits. If a similar ‘mediating effect’ were to exist in adolescent girls, the selection of sedentary girls may dilute the (indirect) relation between 2D:4D and physical fitness in the present study. This however seems very unlikely since it was shown that a subsample of the present study (n = 129) did not differ in terms of digit ratio from age-matched world class gymnasts, in spite of a difference in hours of exercising of approximately 25 hours/week [18], [19].

A final limitation may lie in the use of the left-hand digit ratio. In most studies the digit lengths were taken on the right hand to calculate 2D:4D. This is based on the fact that results of previous studies have suggested that sex differences in 2D:4D and correlations of 2D:4D with target traits are more pronounced for the right hand than for the left hand [30]. Testosterone-dependent physical traits tend to be more strongly expressed on the right side of the body compared to the left side [28], however a meta-analyses focusing specifically on the relation between the 2D:4D digit ratio and athletic prowess demonstrated that neither hand out-predicts the other [20].

The present study is to our knowledge the first to study the relation between 2D:4D digit ratio and multiple distinct components of physical fitness in adolescent girls. The results show that, unlike other anthropometric traits, 2D:4D digit ratio, a putative correlate of prenatal exposure to testosterone, is not related to any physical fitness component in this population. Future studies including adolescent boys should be undertaken to verify if similar results are found. If talent detection in function of elite performance in particular sports is the aim of measuring digit-ratios, future studies should consider testing children and adolescents for this alleged association, since talent detection for sports in adulthood makes no sense. The current results, however, seem to suggest that measurement of digit ratios has no place in a talent detection programme for physical fitness and athletic abilities in adolescent girls.
